# Immunotherapy: Reshape the Tumor Immune Microenvironment

**DOI:** 10.3389/fimmu.2022.844142

**Published:** 2022-07-06

**Authors:** Bingzhe LV, Yunpeng Wang, Dongjiang Ma, Wei Cheng, Jie Liu, Tao Yong, Hao Chen, Chen Wang

**Affiliations:** ^1^ Department of General Surgery, Lanzhou University Second Hospital, Lanzhou, China; ^2^ The Second Clinical Medical College, Lanzhou University, Lanzhou, China; ^3^ Key Laboratory of Digestive System Tumors of Gansu Province, Lanzhou University Second Hospital, Lanzhou, China; ^4^ Department of Surgical Oncology, Lanzhou University Second Hospital, Lanzhou, China

**Keywords:** tumor immune microenvironment, immunotherapy, immune cell, antibody, small molecule inhibitor

## Abstract

Tumor immune microenvironment (TIME) include tumor cells, immune cells, cytokines, etc. The interactions between these components, which are divided into anti-tumor and pro-tumor, determine the trend of anti-tumor immunity. Although the immune system can eliminate tumor through the cancer-immune cycle, tumors appear to eventually evade from immune surveillance by shaping an immunosuppressive microenvironment. Immunotherapy reshapes the TIME and restores the tumor killing ability of anti-tumor immune cells. Herein, we review the function of immune cells within the TIME and discuss the contribution of current mainstream immunotherapeutic approaches to remolding the TIME. Changes in the immune microenvironment in different forms under the intervention of immunotherapy can shed light on better combination treatment strategies.

## Introduction

The immune system can eliminate tumor cells through the cancer-immune cycle (1). This process is not sustained because tumors can gradually shape the tumor immune microenvironment (TIME) into an immunosuppressive state to combat host immunity, and the balance between pro- and anti-tumor inflammatory mediators may determine tumor progression. Tumors have evolved various mechanisms to evade immune surveillance, such as defecting the antigen presentation machinery, enhancing negative immune regulatory pathways, recruiting tumor-promoting immune cells, and others (2–4). The result is that the function of anti-tumor immune cells is blocked, and it is difficult to maintain anti-tumor immune responses. The tendency for antitumor immunity is determined within the TIME by two immune components, antitumor and pro-tumor (5). Despite heterogeneity across different cancer types and populations, the role of the TIME in tumor progression is similar. The goal of immunotherapy is to restore the killing effect of anti-tumor immune cells on tumors, especially cytotoxic T lymphocytes (CTL). However, pro-tumor immune cells, such as regulatory T cells (Tregs) and myeloid-derived suppressor cells (MDSCs), tumor-associated macrophages (TAM), and group 2 innate lymphoid cells (ILC2s), play an important role in impairing anti-tumor immune responses and shaping an immunosuppressive microenvironment. Studying the functions and mechanisms of tumor-promoting immune cells will help to improve the response rate of immunotherapy and develop new immunotherapeutic strategies.

Based on the understanding of tumor immune escape, several cancer immunotherapies have been developed to reshape the TIME to subdue tumor cells. Blocking CTLA-4 and PD-1/PD-L1 immune checkpoints can relieve the functional inhibition of T cells (6). Changing the polarization state from M2 to M1 in TAM (dual blocking of PI3K-γ pathway and CSF-1/CSF-1R) can lead to the reduction of immunosuppressive macrophages and the activation of CD8+T cell response (7). DC-based vaccines can activate T cell responses by removing the inhibition of antigen presentation (8). Therapies that reshape TIME could, in theory, remove tumors through the body’s immune system. This mode has higher specificity and lower side effects, and the generation of memory T cells guarantees a sustained response. Understanding the changes in the TIME during tumor development can help to develop targeted therapeutic strategies and improve response rate of immunotherapy. Recently, new advances have been made in the study of the TIME. This review provides a brief overview of the role of tumor-associated immune cells during remodeling of the TIME. In addition, we introduce the contribution of current mainstream immunotherapy approaches to remolding TIME, with a particular focus on immune cell changes.

## TIME

**Figure 1:**
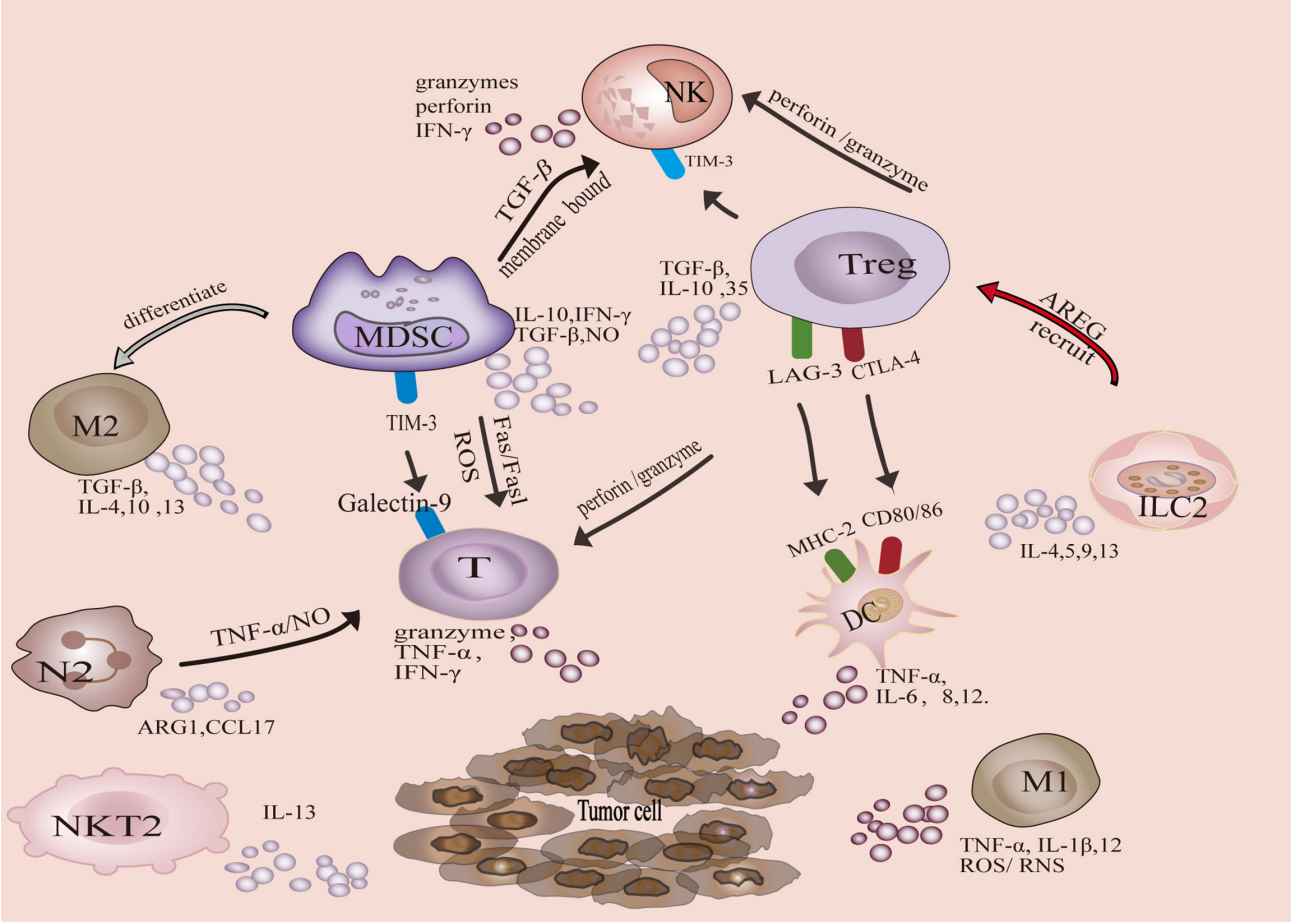
Crosstalk of tumor-associated immune cells in tumor microenvironment.

Tumor-associated immune cells can be divided into two categories, anti-tumor and tumor-promoting. Antitumor immune cells mainly include effector T cells (including cytotoxic CD8 + T cells and effector CD4 + T cells), natural killer cells (NK), dendritic cells (DC), and M1-polarized macrophages. The tumor-promoting immune cells are mainly Tregs, MDSCs, M2-polarized macrophages, N2-polarized neutrophils, natural killer T Type 2 cells (NKT2) cells, and ILC2s (**Figure 1**). In addition, metabolic and biochemical components significantly influence immune cell function.

### Anti-Tumor Immune Cells

T cells are the main executor of anti-tumor immune response, including CTL and T helper cells. CTL recognizes MHC-I molecules expressed by tumor cells (9), exerting tumor killing mechanisms through granule exocytosis (granzyme A and B) or death ligand-induced necrosis and apoptosis under the action of chemokines (10). IFN-γ and TNF-α are secreted to induce tumor cells cytotoxicity (10). CD4+T cells can promote CTL proliferation, increase antigen presentation by DCs, promote CTL activation, and promote memory CTL formation (11, 12).

DCs, as the most potent specific antigen-presenting cells (APCs), initiate adaptive immune responses by activating naive T cells (13). DCs can also express CD80/CD86, which interacts with CD28 to generate costimulatory signals that increase T cell activation (14). DCs produce TNF-α, IL-6, IL-8, and IL-12 to participate in anti-tumor immunity. Tumor cells lose MHC-I molecular to evade immune surveillance by T cells (activation of CD8 + T cells requires MHC-I molecule dependent antigen presentation). The activation of NK cells is inhibited by binding of inhibitory receptors to MHC-I molecules, so NK cells can eliminate targets with defective expression of MHC-I molecules through a “missing self” mechanism (15, 16). NK cell lysis of tumor cells is mainly dependent on granzymes and perforin (17).

The polarization of classically activated macrophages (M1) is mainly mediated by GM-CSF, IL-12, IL-18, IFN-γ, and TNF-α (18, 19). M1 promote Th1 response by secreting TNF-α, IL-1β, and IL-12, and promote the recruitment of Th1 cells to inflammatory sites by secreting chemokines CXCL9 and CXCL10 (20). Besides, M1 macrophages exert antitumor effects through the release of reactive oxygen/nitrogen species (ROS/RNS) directly mediated cytotoxicity and antibody-dependent cell-mediated cytotoxicity (21, 22).

### Tumor-Promoting Immune Cells

Tregs play a key role in maintaining immune homeostasis and peripheral tolerance (23). The physiological function of Tregs is to prevent the spread of inflammation and limit tissue damage, but it acts as a feedback mechanism to inhibit anti-tumor immune response in the TIME. Tregs inhibit anti-tumor immune response through production of immunosuppressive cytokines, such as TGF-β, IL-10 and IL-35 (24). Furthermore, Tregs can inhibit anti-tumor immune responses in several ways:1) Tregs inhibit CTL-mediated tumor killing *via* TGF-β -dependent cell contact (25), promoting polarization of M2 macrophages by inhibiting IFN-γ secretion by CTL cells (26), and inhibiting the generation of memory CD8+T cell through CTLA-4 (27). 2) Tregs inhibit NK cell proliferation, IFN-γ production, degranulation and cytotoxicity, which is related to TIM-3 (28). 3) Treg induces DC functional inhibition in these two ways. Treg-expressed CTLA-4 binds to CD80/CD86 on the DC to down-regulate costimulatory signal (29). Furthermore, MHC class II molecules are the major ligands for LAG-3 (30). LAG3 expressed by Tregs can inhibit the expression of MHC II molecules in DCs (31). 4) MDSCs and Tregs reinforce each other to enhance the immunosuppressive microenvironment. Induction of Tregs can be facilitated by TGF-β, IL-10 and IFN-γ secreted by MDSCs. Tregs enhance the function of MDSCs through TGF-β and IL-35 (31). 5) Tregs can induce NK and CD8+T cell death in a granzyme B and perforin dependent manner (32, 33).

MDSCs represent a heterogenous population of immature myeloid cells with different transcriptional activities and differentiation states, characterized by immunosuppressive activity in pathological states (34). MDSCs can be roughly divided into two groups, granulocytic or polymorphonuclear MDSCs (PMN-MDSCs) and monocytic MDSCs (M-MDSCs). PMN-MDSCs can produce ROS and reduce T cells responses to antigens (34). PMN-MDSCs induce CTL apoptosis through the Fas/FasL axis, whereas M-MDSCs produce nitric oxide to inhibit immune activation (35). M-MDSCs can also differentiate into immunosuppressive macrophages and inhibit T cell activation (36). In addition to interacting with Tregs, IL-10, and TGF-β produced by MDSC also impair CTL function (37). MDSCs reduce NK cell numbers and inhibit their function *via* membrane-bound TGF-β (38). MDSCs express galectin 9, which binds to TIM-3 on lymphocytes and induces T cell apoptosis (39). Inhibition of MDSCs enhances the function of T cells (36, 40).

M2 macrophages are usually the dominant cells in TAM. M2 macrophage polarization is mediated by M-CSF, IL-4, IL-10, IL-13 and TGF-β (41). M2 macrophages secrete immunosuppressive cytokines, TGF-β, IL-4, IL-10 and IL-13 (19, 42–44). M2 macrophages are involved in activating Th2 immune response (45). In addition, M2 macrophages, involved in the recruitment of Tregs cells *via* M2 derived CCL20/CCL22 (46), as well as by increasing the expression of PD-L1 to attenuate the effects of CTLs and induce MDSC differentiation (47, 48).

Polarization of N2 neutrophils is mainly mediated by TGF-β (49). Recently, IL-6 produced by gastric cancer mesenchymal stem cells was also found to determine N2 polarization (50). N2 neutrophils induce CD8 +T cells apoptosis through TNF-α and NO-dependent mechanism (51). In addition, N2 can also inhibit T cell proliferation by releasing argininase-1 (ARG1) and regulating PD-L1/PD-1 signaling (52), as well as secreting chemokine CCL17 to recruit Tregs (53). Although the exact mechanism remains nonclear, studies have shown that N2 neutrophils inhibit NK cell function (54).

ILC2 secrete cytokines (IL-4, IL-5, IL-9, IL-13) and recruit immunosuppressive cells to shape the TIME (55). ILC2 secrete IL-13, which promotes the aggregation of MDSC and inhibits the anti-tumor response of CTL (56, 57). Besides, ILC2 induce the production of TGF-β from MDSCs, which contributes to the polarization of M2 macrophages (58). LC2s produce the epidermal growth factor (EGF)-like molecule Amphiregulin (AREG), which costimulates ICOSL/ICOS to establish and maintain an immunosuppressive microenvironment, leading to Treg activation and accumulation (59, 60). In addition, ILC2s may inhibit the activity of NK cells (61).

NKT switch between inflammatory and immunosuppressive subsets to respond to the TIME status (62). NKT1 is antitumor, while NKT2 is primarily tumor-promoting.IL-13 produced by NKT2 induces MDSC to produce TGF-β, which inhibits the anti-tumor immune response mediated by CD8+T cells (63). In myeloma, the weakening of NKT2 cell population has the potential to mediate tumor regression (64).

### Metabolic: Hypoxia-Adenosinergic Immunosuppression

The numerous and complex cell populations and the limited vasculature within the tumor microenvironment render nutrient and oxygen delivery and waste clearance inefficient. In addition, tumor cells shape the metabolic fitness of tumor infiltrating immune cells by competing for and consuming essential nutrients or otherwise, such as the classical ‘Warburg effect’. Tumors prefer to perform aerobic glycolysis to convert virtually all glucose to lactate even in the presence of oxygen (65). Metabolism in the microenvironment, such as nutrient consumption, increased oxygen consumption, and production of reactive nitrogen and oxygen intermediates, significantly influences antitumor immune responses. As a result, high lactate and low pH, hypoxia, and high levels of ROS are prevalent in the TME. This hostile environment shapes the metabolic adaptation of tumor infiltrating immune cells, and these metabolic changes in immune cells undermine the effectiveness of antitumor immune responses. (A more detailed overview of immunometabolism in review (66, 67)). The main focus here is on the role of the hypoxia adenosine in immunosuppression as well as adenosinergic blockade in reprogramming the TIME. Tumor, especially the solid tumor microenvironment, provides fertile soil for adenosine production. A series of cascades driven by the hypoxia/HIF-1α-CD39/CD73 axis represent major sources of adenosine (68, 69). In addition, some alternative activation modalities, CD38, CD203a, and PAP also contribute to adenosine levels in the TIME (70, 71). There are four receptors for extracellular adenosine, A1, A2a, A2b, and A3. Adenosine is an immunosuppressive metabolite, signaling largely through the A2a receptors on innate and adaptive immune cells (72). A2AR is upregulated due to hypoxia induced HIF-1α transcriptional activity (73). Adenosine accumulation in the TIME can inhibit antitumor immune cell functions by binding to A2AR. For example, T cells and NK cells (68, 74, 75). In addition, adenosine enhances the activity of immunosuppressive cells, such as MDSCs and Tregs, contributing to CAF shaping as well as inducing the formation of new blood vessels (76–80).

## Therapy to Reshape TIME

**Figure 2:**
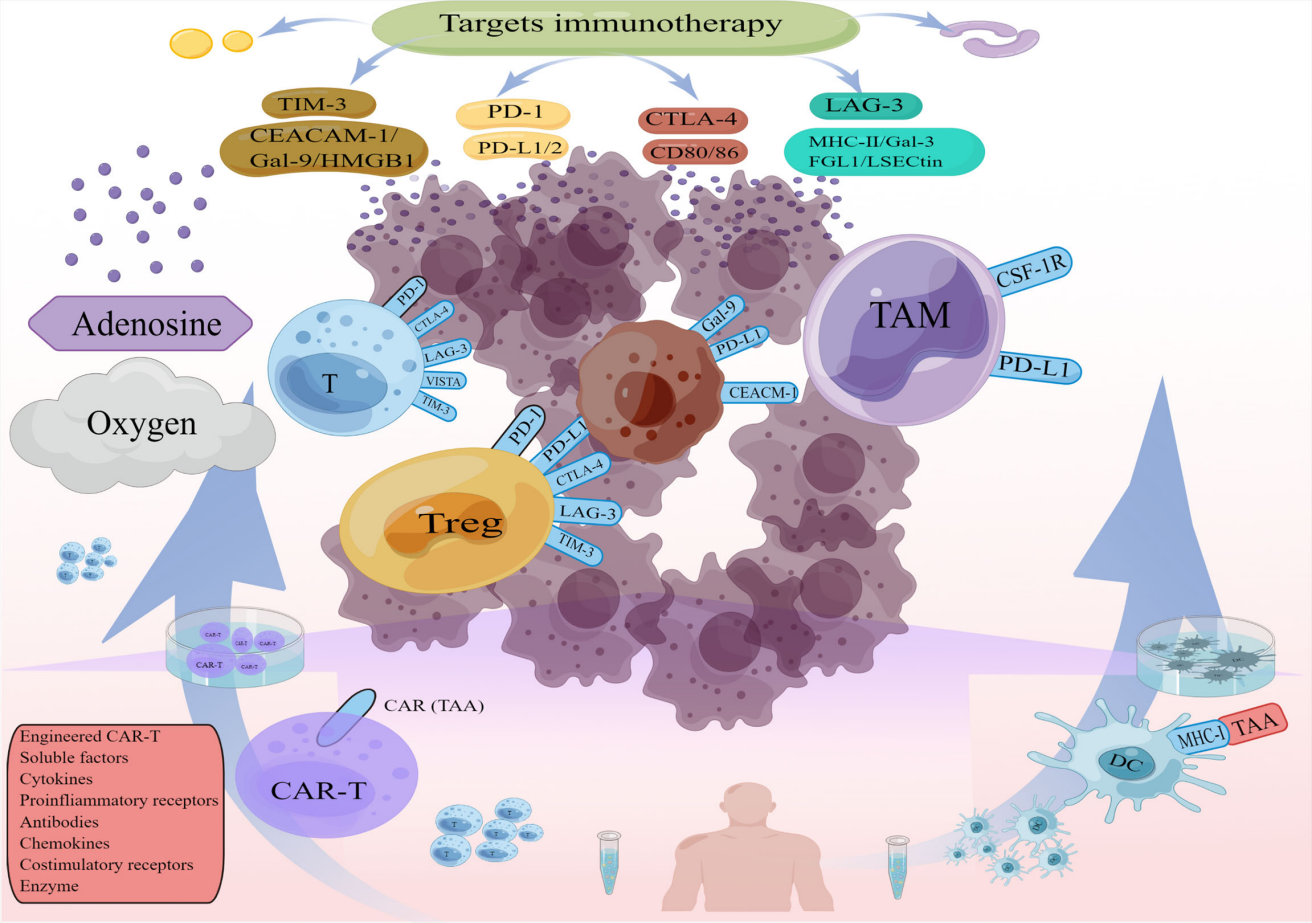
Crosstalk of various treatments in TIME (By Figdraw).

Currently mainstream immunotherapies, Immune checkpoints, CAR-T, DC cell vaccines, contribute to reshaping the TIME, (e.g., changes in immune cells and cytokines after immune checkpoint blockade, changes in T cell function and microenvironmental status after CAR T infusion). This has driven the development of combination therapies. The effects of different immunotherapies on the TIME help us to find effective combination treatment strategies (**Figure 2**). For example, most classically, CAR T cells provide infiltration while Immune checkpoints inhibitors (ICIs) reverse CAR T cells inhibition and restore functional persistence.

### Targets for Antibodies and Small Molecule Inhibitor

At present, the mainstream strategy of immunotherapy is to target some components of the TIME through antibodies or small molecule inhibitors (**Table 1**). We will summarize the key functions of major immunotherapies in the TIME, with an emphasis on how therapeutically enabling the TIME to generate an anti-tumor immune response. During anti-tumor immunity, negative regulators of T cell activation act as “checkpoint molecules” to modulate the immune response. Depending on checkpoints. This strategy can be implemented by inhibiting inhibitory checkpoints or activating stimulant checkpoints. CTLA-4 and PD-1 are the most effective examples of immune checkpoint therapy.

**Table 1:** Immunotherapy targets within TIME and the treatment effect.

Targets	Treatment Effects on TIME	Reference
CTLA-4	Promote the reduction of Treg cells, enhance the activity of effector T cells	(29, 81–84)
PD-1/PD-L1	1. Promote the expansion and migration of CD8+ T cells and inhibit their apoptosis. 2. Amplification of CD4 + T cell subsets. 3. Proliferation, survival and activation of macrophages (co-stimulating molecular expression, cytokine production), and enhancement of phagocytosis of macrophages. 4. Activation of NK cell response, especially in the NK cell subpopulations (CD69 and SCA-1).	(85–89)
LAG-3	Increased populations of APC, NK and CD8 + T cells,	(90, 91)
TIM-3	1. Promote antibody-dependent phagocytosis of bone marrow cells/macrophages expressing FcγR and promote the M1 phenotype. 2. Enhance DC antigen presentation ability and promote DC maturation. 3.CD8+T cell proliferation ↑, IFN-γ production ↑. 4.Treg proliferation ↓, cell apoptosis ↑	(92–97)
TIGIT	1. Proliferation, cytokine production and cytotoxicity of CD8 + T cells. 2. Depletion of Tregs and proliferation of CD4 + T cells. 3. Inhibits the depletion of tumor-infiltrating NK cells.	(98–100)
VISTA	Improve the infiltration, proliferation and effector function of tumor-infiltrating T cells in TME.	(101)
Siglec-15	Reverse TAM-related inhibition of T cell activity.	(102)
CSF-1/CSF-1R	1.Reduce TAM and induced residual TAM to polarize M2 phenotype, increase the level of tumor-infiltrating lymphocytes. 2.Increased PD-1/PD-L1 expression on TAM and CTLA-4 expression on CD8+ T cells.	(103, 104)
FAK	Decrease immunosuppressive MDSC, TAM and Treg, increase CD8+ T cells and enhance CD8+ T cell-mediated antitumor activity in tumors.	(105–107)
TGF-β and isoform	Increase the number of CD8+ T cells, establish immunological memory in TIME and decrease immunosuppressive myeloid cells.	(108, 109)
VEGF-A	Decrease PD-1 expression of CD8+ T cells.	(110, 111)

CTLA-4 is induced on the cell surface of conventional T cells by antigen activation and is constitutively expressed on Treg (85). As having the same B7 ligands as CD28, including B7-1 (CD80) and B7-2 (CD86), CTLA-4 with a higher affinity competes with CD28 expressed on effector T cells for binding to B7-1 and B7-2 which ubiquitously expressed on B lymphocytes, dendritic cells, and other immune cells (112). The result is that costimulatory signals CD80/CD86 which can activate T cells by ligating CD28 are inhibited. As a result, T lymphocyte proliferation and cytokine secretion are hampered (113). CTLA-4 can reduce the activation of T cells by generating inhibitory signals, thereby attenuating the anti-tumor immune response. CTLA-4 can induce indoleamine 2,3-dioxygenase (IDO) and trigger reverse signaling through B7 ligands to inhibit T cell proliferation (114). Inhibition of CTLA-4 enhanced the antitumor activity of effector T cells mainly by inhibiting Treg. Anti-CTLA-4 antibody enhances IL-36 stimulated anti-tumor activity by consuming Tregs, leading to increased CD4+ and CD8+T cells proliferation and IFN-γ levels (115). Anti-CTLA-4 antibody reduces Treg cells in the TIME but do not affect the status of peripheral lymphoid organs, reducing immune-related adverse events (irAEs) (116). Compared with glycoprotein 100 (gp100) peptide vaccine, melanoma patients treated with ipilimumab had improved survival (OS of 10 months/ipilimumab and 6.4 months/GP100, P<0.001) (117). A pooled analysis of 10 prospective studies and 2 retrospective studies, including 1,861 patients with advanced melanoma, found a 22% 3-year survival rate in patients treated with ipilimumab (118).

PD-1 is a transmembrane protein mainly expressed on the surface of activated T cells, B cells and macrophages. PD-L1 and PD-L2 are dual ligands of PD-1, and both have been shown to inhibit T cell activity upon PD-1 engagement (119). PD-L1 over-expressed on cancer cells can interact with PD-1 on activated T cells, inducing T cell inhibition and CTL dysfunction. Correspondingly, blockade of PD-1/PD-L1 promotes pro-inflammatory factors release, T cells proliferation, CTL activation (120). PD-1/PD-L1 is an ideal immunotherapeutic target to restore the effector function of anti-tumor specific T cell. Patients with metastatic melanoma who respond to anti-PD-1 therapy (pembrolizumab) exhibit active CD8+T cell proliferation in the TIME, which is associated with reduced tumor size (121). In addition, it has been observed in melanoma patients that CD4 + T cells expand after PD-1 blockade and that activated CD4 + T cells secrete IFN- γ and chemokines, which contribute to antitumor immunity (86). A trial that enrolled 655 patients with advanced or metastatic melanoma showed long-term antitumor activity and tolerability of pembrolizumab in advanced melanoma, with a mean follow up of 55 mouths. The estimated 5-year overall survival rate was 34% for all patients, 41% for patients receiving initial treatment (122). Pembrolizumab also provided a long-term response and prolonged OS in non-small cell lung cancer, with the combination of pembrolizumab and chemotherapy achieving objective response in 55% of patients compared to 29% of who those treated with chemotherapy alone, with a significantly longer median PFS than chemotherapy (13.0 months vs 8.9 months) (123). Blockade of PD-1/PD-L1 can restore the killing ability of T cells and induce tumor regression, resulting in better clinical outcomes (119).

LAG-3 is expressed on tumor-infiltrating T cells with defective cytokine production and on Tregs (124, 125). Treg cells with high expression of LAG-3 produce immunosuppressive cytokines IL-10 and TGF-β and inhibit effector T cell activity (126). LAG-3 expression levels correlate with tumor progression and poor prognosis (126). Anti-LAG-3 antibodies slow tumor growth in mouse model of fibrosarcoma (127). The combination therapy of anti-LAG-3 antibody and tumor-associated antigen inoculation increases CD8+T cells in the TIME and destroyed tumor parenchyma in prostate cancer tumor models (128). LAG-3 and PD-1 were highly co-expressed in CD4+T cells and CD8+T cells, and the inhibitory effect of the blocking of LAG-3 and PD-1 on tumor progression (129, 130). Moreover, dual blockade of LAG-3 and PD-1 can also increase the number of tumor-infiltrating CD8+ T cells and reduce Treg, thereby synergically enhancing anti-tumor immunity (131). In a Phase I/II study (NCT0198609) evaluating the safety and efficacy of anti-LAG-3 antibody in combination with anti-PD-1 antibody, 61 melanoma patients in a Phase I/II study well tolerated with an ORR of 11.5%. Patients with high LAG-3 expression had a significantly higher objective response rate than those with low expression (132, 133). PD-L1/LAG-3 bispecific antibody induced stronger anti-tumor effect than each parental antibody (134, 135). In addition, PD-1 and LAG-3 blockade improve anti-tumor vaccine efficacy (136). As mentioned above, there is a synergistic effect between anti-LAG-3 and certain immunotherapies, and the combination of anti-LAG3 with more therapies is worth investigating. According to the present findings, LAG-3 is a promising cancer therapeutic target secondary to PD-1/PD-L1 and CTLA-4.

T cell immunoglobulin and mucin-domain containing-3 (TIM-3), T cell immunoglobulin and ITIM domain (TIGIT), V-domain immunoglobulin suppressor of T cell activation (VISTA) and Siglec-15 are also attractive targets (**Table 2**). Cancer immune checkpoint therapies are increasingly being developed to restore immune activity against tumor cells. In addition to monotherapy, rational combination of immunotherapy and multi-target combination such as bispecific antibodies, trispecific antibodies have also been considered to achieve synergistic effects to inhibit tumor growth. We anticipate that translating candidate targets into the clinical area may yield better clinical benefits than CTLA-4 or PD-1 inhibitors.

**Table 2:** Functions of new immune targets.

Targets	Cells expressing	Ligands	Mechanism	References
TIM-3	Th1, Th17, CD8T, Treg, NK, DC	Galectin-9, HMGB1, CEACAM-1, PtdSer	1.Binds Gal-9 to induce apoptosis in Th1 and CD8 TIL. 2. TIM-3 blockade enhance tumor antigen-specific T-cell proliferation and activity. 3.TIM-3 interacted with HMGB1 to suppress antitumor immunity mediated by nucleic acids	(137–145)
TIGIT	CD8 T, CD4 T, NK, Treg	CD155, CD112, CD113	1.TIGIT indirectly impedes T cell function by binding to CD155 on DCs, promoting tolerogenic DCs with decreased production of IL-12 and increased production of IL-10. 2.TIGIT exhibits direct immune cell-intrinsic inhibitory effects. 3. TIGIT inhibits NK cell degranulation, cytokine production, and NK cell-mediated cytotoxicity of CD155-expressing tumor cells. 4.TIGIT is highly expressed on Tregs and TIGIT+Tregs demonstrated to be superior in suppressing T cells.	(146–153)
VISTA	T cell, Treg, macrophage, myeloid cell subset	PSGL-1, VSIG3	1.over-expression of VISTA suppressing T cell immunity 2. Anti-VISTA reduces the number of MDSCs and tumor specific Tregs, increases the proliferation of TIL and promotes T cell effector function. 3.VSIG3 interaction with VISTA on T cells suppresses T cell activation and proliferation.	(154–158)
Siglec-15	macrophage,	Sialyl-Tn	1.Siglec-15 ablation slowed down tumor growth and prolonged survival. 2. A expansion of tumor-infiltrating CD8+ T and NK cells as well as several inflammatory myeloid populations, whereas a decrease of TAMs and MDSCs.	(159, 160)

Small molecule drugs, which are more suitable for oral administration than polymeric antibody drugs, could reduce severe immune-related adverse events (irAEs) resulting from prolonged target occupancy by modulating the half-life of the drug (161, 162). They can cross the cell membrane, penetrate more easily into the tumor tissue and aggregate in a sufficient concentration (161, 162). In addition, they are lower production costs and higher stability (163, 164). We focus here on the role of colony stimulating factor-1 receptor (CSF-1R) in reshaping TIME. CSF-1/CSF-1R signaling is a key activator of the mononuclear phagocyte system, and blockade of CSF-1/CSF-1R creates an environment of reduced immunosuppression and enhanced interferon response that can impede tumor growth (103). Pexidartinib (CSF-1R inhibitor) was demonstrated to alter the distribution of TAMs in TIME and reduce tumor volume in a mouse model of lung adenocarcinoma (165). In a mouse model of BRAF V600E mutant melanoma, Pexidartinib combined with adoptive cell transplantation decreased TAM and increased tumor-infiltrating lymphocyte levels (166). The combination of Pexidartinib and BRAF inhibitors resulted in a significant inhibition of tumor growth by reducing the recruitment of M2 macrophage (104). Moreover, in a pancreatic cancer mouse model, blocking of CSF-1/CSF-1R reduced M2 macrophages within the TIME and polarized the remaining TAMs into an anti-tumor phenotype (103). This study also found that PD-1/PD-L1 expression on TAMs and CTLA-4 expression on CD8+ T cells was increased in the presence of CSF-1/CSF-1R blockade, and the combination of PD-1 or CTLA-4 antagonists resulted in more significant tumor regression (103). A recent clinical study of advanced tenosynovial giant cell tumor found that Pexidartinib significantly reduced tumor size with an overall response rate of 39% (167). To enhance the response of CSF-1/CSF-1R blockade, researchers tend to combine CSF-1/CSF-1R blockade with immunotherapy. The combination of oncolytic virus, CSF-1R inhibition and anti-PD-1 immunotherapy was found to enhances anti-tumor immune response by increasing T cell infiltration and augmenting anti-tumor CD8+ T cell function (168). In addition, the combination of targeted TIME and anti-angiogenesis has been suggested to enhance the antitumor activity of the drug (169). For example, the combination of the tyrosinase inhibitor sorafenib and the immunomodulator lenalidomide has long been found to be more effective than drugs alone (170). Surufatinib is a drug that targets tumor angiogenesis (VEGFR and FGFR1) and tumor immune evasion (CSF-1R). Surufatinib was effective against neuroendocrine tumors in two phase III trials. Whereby it may become a mainstream treatment for neuroendocrine tumors (171). These results suggest that in addition to developing drugs for novel targets, multitarget fusion drugs or combinations of antibodies and small molecule immune modulators also play important roles in reshaping TIME.

Despite the success of anti-PD1/PD-L1 and anti-CTLA4 therapies in advanced cancer. There are a considerable number of patients who remain unresponsive or relapses after initial response. Combination strategy of immunotherapy are used to address these challenges. Furthermore, targeting hypoxic adenosine pathway represents another idea to improve immunotherapy (172, 173). This treatment can be broadly divided into two types.

1. Reduced adenosine production. As mentioned above, hypoxia induces upregulation of CD39 and CD73 and downregulation of adenosine transporters to promote the accumulation of extracellular adenosine. Hyperoxic respiration (60% O2) significantly reduced adenosine levels and gained tumor control and prolong survival. Hyperoxic breathing upregulates antigen-presenting MHC class I molecules on tumor cells, tumor infiltration of CD8 T cells and attenuate immunosuppressive effects of Tregs (69, 174). CD73 can convert AMP produced by catabolism to adenosine. Antagonism of CD73 increases the activity of CD8 + T cells, B cells and related cytokine, and tumor growth and metastatic spread are retarded in CD73 blocked mice (175–177). Compared with anti-PDL1 alone, ORR close to 40% in the dual CD73/PDL1 blockade arm with statistically improved 10-month PFS (64.8 vs 39.2) (NCT03822351). Similar to studies with CD73, tumor growth and metastasis were reduced in CD39-blocked mice (178, 179). CD39 blockade enhance the function of T, NK cells, as well as decreased Treg-mediated immunosuppression (178, 180, 181). Finally, although studies have demonstrated the effectiveness of this approach within tumor-bearing mice. Clinical studies exploring CD39 blockade/inhibition have not yet yielded results.

2. Block the binding of adenosine receptor. A2AR antagonists are a more direct approach to inhibit adenosine induced signal transduction. A2AR-deletion leads to delayed tumor progression and prolonged survival (68, 182). TIME of A2AR antagonist treated mice showed similar changes in immune level as blockade of CD39/73, which was more infiltrated by CD8 T cells and NK cells and contained fewer Tregs (183, 184). Besides, A2AR blockade reduced PD-1 and LAG-3 expression on Tregs and T cells (182). For renal cell cancer, A2AR antagonism (CPI-444) induces durable responses when used as monotherapy as well as in combination with anti-PD-L1. Patients who experienced a positive response included individuals who were resistant or refractory to anti-PD-1/PD-L1 antibodies. Adenosinergic blockade resulted in higher cytotoxic T cell tumor infiltration (172). Besides, A2AR antagonists have shown similar activity in other types of cancer (NCT02403193, NCT03720678, NCT03720678).

### Adoptive Cell Therapy

Adoptive cell therapy (ACT) uses autologous immune cells that are isolated, engineered, amplified and injected into a patient to generate durable anti-tumor immune response. T cells genetically modified to express chimeric antigen receptor (CAR), or CAR T, are the most effective cell therapies. CAR T cells specifically recognize tumor-associated antigens (TAA) and kill tumor cells (185). Adoptive transfer of tumor-reactive T cells resulted in persistent clonal repopulation of T cells in patients, with the transferred cells proliferating, displaying functional activity, trafficking to the tumor sites and promoting tumor control (186). Anti-CD19 CAR T cell can produce cytokines that respond specifically to CD19+ target cells and effectively eradicate lymphoma cells (187). In a clinical trial, 82% (89/108) of patients with refractory large B-cell lymphoma (ZUMA-1) achieved an overall response and 58% (63/108) achieved a complete response (188). CAR T cells have achieved remarkable success in treating hematologic malignancies but face unique challenges in solid tumors, such as lack of suitable targets, inefficiency of CAR T cells to infiltrate into tumor sites, and TIME limitations on CAR T efficacy (189). CAR-T holds promise in addressing these issues through diversifying edits and in combination with other therapy approaches. Her-2, a receptor tyrosine kinase overexpressed in many human cancers, is used as a TAA for targeted CAR T in glioblastoma. Although such CAR T cells only expand for a short term, they maintain long-term antitumor activity (190). CAR T cells expressing high levels of the CCR2 receptor can migrate more efficiently to CCL2-secreting tumor sites and exhibit greater antitumor activity (191). In addition, overexpression of heparanase (HPSE), an enzyme that can degrade major components of the extracellular matrix, on CAR T cells effectively promotes tumor T-cell infiltration and antitumor activity (192). CAR-T editing is diverse, for example, PD-1 knockdown can inhibit immune checkpoint signaling (193), LAG-3 knockdown can disrupt negative regulators of T cell activity (194), IL-12-secreting CAR T cells polarized TAMs to M1 phenotype and reduce the levels of MDSCs and Tregs in mouse models (193), IL-18-secreting CAR T cells can increase M1 macrophages, activated DC and activated NK cells while decreasing M2 macrophages and Treg in the TIME (195). CAR T cells co-expressing CCL19 and IL-7 can recruit large numbers of endogenous T cells and DCs to enhance and sustain tumor clearance (196). In addition to the specific operation of CAR T cells, the therapy of reshaping the TIME can theoretically enhance the therapeutic effects of CAR T cells (197).

### Cancer Vaccines

Cancer vaccines generate antitumor immune responses against TAAs or tumor-specific antigens (TSAs). DCs are specialized antigen-presenting cells that are key targets for cancer vaccines because of their unique ability to link innate and adaptive immunity (198). The main route of this strategy is to modulate the antigen presenting function of DCs to enhance the antitumor immune response in the TIME. Sipuleucel-T, composed of cultured peripheral blood mononuclear cells containing activated APCs, induces sustained responses of T and B cells to the target antigens prostatic acid phosphatase (PAP) and GM-CSF (199). Patients with localized prostate cancer, when Sipuleucel-T was administered preoperatively, exhibited increased T-cell proliferation and IFN-γ levels. In addition, infiltrating T cells were increased more than threefold in resected tissue specimens after surgery compared with controls (P 0.001) (200). In the early stages of prostate cancer, Sipuleucel-T significantly promotes the activation of APCs and increases the level of antigen presentation (201). In a clinical trial for metastatic prostate cancer (NCT00065442), Sipuleucel-T significantly improved OS compared with placebo (median 25.8 months vs 21.7 months, [HR] 0.78, P =0.03) (202). This suggests that cancer vaccines have considerable potential in tumor immunotherapy. In phase I clinical trial of nine men with metastatic castrate-resistant prostate cancer treated with Sipuleucel-T and escalating doses of ipilimumab showed that IgG and IgM levels against PA2024 and PAP increased significantly after ipilipumab (203). Combination therapy with vaccines and checkpoint inhibitors is effective, although few vaccines are now available for clinical use and multiple clinical studies have been negative (204–207). Vaccines can trigger long-term immunological memory, thus contributing to long-lasting anti-tumor immune response. Antigen and vaccine vectors were developed to achieve optimal antigen presentation by APCs, combined with multiple approaches to overcome immune evasion and immunosuppression by cancer cells. Development of antigens and vaccine vectors to achieve optimal antigen presentation by APCs, as well as combination therapy approaches, hold promise to overcome immune evasion and immunosuppression by cancer cells.

## Combination Therapy

Different treatments reshape TIME in different ways. Therefore, we can combine complementary or augmentation strategies to achieve better clinical outcomes. Herein, we discuss the principles and clinical application of combination therapy.

CAR T cells can provide an infiltrate for the TIME, and ICI can reverse CAR T-cell inhibition and restore functional persistence. CAR-T may escalate the expression of PD-1 inhibitory signaling, and interference of PD-1 pathway may restore the effector function of CAR-T cells (208). Combination therapy of oxaliplatin and anti-PD-L1 synergistically improves CAR-T cell-mediated lung tumor control and survival (193, 209, 210). Patients with Malignant Pleural Disease in a Phase I Trial of CAR T-cell combination with Pembrolizumab, have a median overall survival of 23.9 months (211). In another multi-center phase II trial, the combination of anti-PD-1 antibody enhanced CAR-T therapy in lymphoma patients with minimal toxicities (212).

Blocking CSF-1/CSF-1R alone increased PD-1/PD-L1 expression on TAM cells and CTLA-4 expression on CD8+ T cells, whereas combination with PD-1 or CTLA-4 antagonists led to more significant T cell infiltration and tumor regression (213–216). CSF-1R inhibitor overcome the resistance to PD-1/PD-L1 axis blockade in an esophageal adenocarcinoma model, resulting in enhanced T cells infiltration and reduced M2 macrophage polarization in the TIME. It confirms that the direct translation of TAM suppression into clinical benefit (217). Emactuzumab, an anti-CSF-1R antibody, has a manageable safety profile in combination with atezolizumab over atezolizumab monotherapy. The increase in CD8 +TILs after therapy appeared to be associated with persistence of TAM subsets (218). Similarly, combined blockade of different targets is worth exploring its clinical effect. Sabatolimab, an Anti-TIM-3 Antibody, in combination with spartalizumab, an Anti-PD-1 Antibody, shows preliminary signs of antitumor activity (219). The combination of relatlimab, a LAG-3-blocking antibody, and nivolumab has been shown to be safe and to have antitumor activity in patients with previously treated melanoma. The ORR was 33% in some pembrolizumab-refractory patients and 50% in PD-1 naïve patients (220). Afterwards, in patients with previously untreated metastatic or unresectable melanoma, this combination did not show a new safety signal but it provided a greater benefit in progression-free survival than inhibition of PD-1 alone (221).

Vaccines will increase tumor-specific T cells due to intensified immunogenicity. Tumor-specific T cells will still be subject to the immunosuppressive microenvironment, which can be altered by checkpoint inhibitors. Effective vaccines combined with therapies targeting the TIME, such as checkpoint inhibitors, are likely to yield optimal results (222). The personalized neoantigen-based vaccine, NEO-PV-01, combined with nivolumab stimulate durable neoantigen-specific T cell responses in patients with advanced melanoma, non-small cell lung cancer, or bladder cancer (207). GX-188E in combination with pembrolizumab showed preliminary anti-tumor activity in patients with recurrent or advanced cervical cancer (223).

Multiple non-redundant immunosuppressive mechanisms coexist within the tumour microenvironment. A major immunosuppressive mechanism is the hypoxia adenosinergic immunosuppressive pathway, which now represents an attractive new target for cancer therapy. Several strategies described above can inhibit this mechanism. The ultimate goal of these strategies is to attenuate hypoxia driven and CD39/CD73 mediated accumulation of extracellular adenosine and immunosuppressive signals (174, 224, 225). This liberates the anti-tumor immunity of T and NK cells. In addition to the combination of A2AR inhibitor and anti-PD-1/PD-L1, A2AR inhibitor was also combined with nanovaccine to activate CD8 T and NK cells and inhibit the proliferation of regulatory T cells. Thus, this strategy could trigger a robust systemic antitumor immune response (173, 226). Furthermore, deletion of A2AR enhances the efficacy of CAR T cells (226, 227). Another way to implement this strategy is hyperoxygenation to improve cancer immunotherapies (69, 224).

In addition to the combination of immunotherapy above, the combination of immunotherapy with antiangiogenics, chemotherapy and radiation is under clinical consideration. Nearly every targeted therapy proven to modulate the immune response is currently being tried in combination with immunotherapy (228, 229).

## Conclusion

The development of immunotherapy has achieved great clinical results, but the heterogeneity of the TIME makes it difficult to determine the best immunotherapy for individuals. There are still many obstacles in the potential development of immunotherapy. The formation of immunosuppressive microenvironment promotes tumor immune escape and restricts the clinical effect of immunotherapy. The further understanding of the TIME mechanism is conducive to the development of immunotherapy. Combined therapy is more conducive to the remodeling of microenvironment and can bring better clinical benefits. However, this raises the question whether improving anti-tumor immunity will lead to more serious irAEs (More detailed explanations in review (66, 230, 231). On the one hand, the further understanding of microenvironment mechanism is expected to balance the internal environment balance between anti-tumor immunity and Irae. On the other hand, the interpretation of a large number of clinical results, including the detection and summary of adverse immune events, helps to determine the best treatment combination.

## Author Contributions

Conceptualization, BL and HC. Writing original manuscript, BL and YW. Visualization, BL. Writing review and editing, BL, YW, DM, WC, JL, TY, CW, and HC. All authors have read and agreed to the published version of the manuscript.

## Funding

This research was funded by the Key Project of Science and Technology in Gansu province (grant no. 19ZD2WA001).

## Conflict of Interest

The authors declare that the research was conducted in the absence of any commercial or financial relationships that could be construed as a potential conflict of interest.

## Publisher’s Note

All claims expressed in this article are solely those of the authors and do not necessarily represent those of their affiliated organizations, or those of the publisher, the editors and the reviewers. Any product that may be evaluated in this article, or claim that may be made by its manufacturer, is not guaranteed or endorsed by the publisher.
